# Exotic skyrmion crystals in chiral magnets with compass anisotropy

**DOI:** 10.1038/srep29126

**Published:** 2016-07-05

**Authors:** J. P. Chen, Dan-Wei Zhang, J. -M. Liu

**Affiliations:** 1 Laboratory of Solid State Microstructures and Innovative Center of Advanced Microstructures, Nanjing University, Nanjing 210093, China; 2 School of Physics and Electronic Engineering, Guangzhou University, Guangzhou 510006, China; 3 Guangdong Provincial Key Laboratory of Quantum Engineering and Quantum Materials, SPTE, South China Normal University, Guangzhou 510006, China; 4 Institute for Advanced Materials, South China Normal University, Guangzhou 510006, China

## Abstract

The compass-type anisotropy appears naturally in diverse physical contexts with strong spin-orbit coupling (SOC) such as transition metal oxides and cold atomic gases etc, and it has been receiving substantial attention. Motivated by recent studies and particularly recent experimental observations on helimagnet MnGe, we investigate the critical roles of this compass-type anisotropy in modulating various spin textures of chiral magnets with strong SOC, by Monte Carlo simulations based on a classical Heisenberg spin model with Dzyaloshinsky-Moriya interaction and compass anisotropy. A phase diagram with emergent spin orders in the space of compass anisotropy and out-of-plane magnetic field is presented. In this phase diagram, we propose that a hybrid super-crystal structure consisting of alternating half-skyrmion and half-anti-skyrmion is the possible zero-field ground state of MnGe. The simulated evolution of the spin structure driven by magnetic field is in good accordance with experimental observations on MnGe. Therefore, this Heisenberg spin model successfully captures the main physics responsible for the magnetic structures in MnGe, and the present work may also be instructive to research on the magnetic states in other systems with strong SOC.

Spin-orbit coupling (SOC) plays an important role in condensed matter physics, especially in recently addressed quantum spin Hall effects and related topological orders^1^,^2^. So far, magnetic materials with lacking inversion symmetry in B20 cubic crystal structure provide an alternative arena for studying the emergent phenomena in magnets of strong SOC. Prominent members encompass metallic compounds like MnSi^3^,^4^,^5^ and FeGe^6^, semiconductor Fe_1–*x*_Co_*x*_Si^7^,^8^, and multiferroic insulator Cu_2_OSeO_3_^9^. In these materials, the competitions between strong ferromagnetic (FM) exchanges, weak Dzyaloshinsky-Moriya (DM) interactions, and additional magnetic anisotropies originating from high-order interactions, generate a long-wavelength helical spin order which propagates along some specific directions favored by these magnetic anisotropies^3^,^4^,^5^.

The roles of magnetic anisotropies are usually delicate but substantial in determining the magnetic ground states in some cases. Generally, these anisotropy terms are the weakest in energy scale among the above mentioned interactions, and the favored directions of helical spin orders are materials-dependent. For example, the helical order in MnSi observed in neutron scattering experiments is characterized by a set of Bragg peaks situated on a sphere surface, suggesting that the spin alignment of the helical state is locked along the cubic space diagonal <111> directions, due to the effect of high-order crystal anisotropies^3^,^4^,^5^. On the other hand, a perpendicular external magnetic field may transform the single twisted helice to the energetically favorable hexagonal skyrmion crystal (SkX) structure^4^,^8^. This intriguing topological structure has been receiving tremendous interest due to its unusual magnetic and transport properties, especially the topological Hall effect^10^,^11^,^12^.

Nevertheless, recent experiments on B20-type cubic MnGe^11^,^13^ revealed the field dependence of the topological Hall effect which is distinct from those observed in other B20-type compounds, suggesting a zero-field ground state skyrmion lattice very different from often observed hexagonal SkX. So far, no recognized theory for this unusual skyrmion lattice is available. In addition, the helical wavelength *λ* changes from 3 nm to 6 nm with temperature, which is much smaller than that in conventional B20-type compounds with typical *λ* of 18–90 nm. Therefore, a different mechanism for the spin structure in this generic B20 MnGe is expected. One possible interpretation of the variation in small-angle neutron scattering (SANS) patterns with magnetic field is the presence of an easy axis of magnetization along the <100> direction^13^. This implies that MnGe may have strong SOC and consequent non-negligible and high-order magnetic anisotropy due to SOC, so that the modulation vector ***q*** is locked along the <100> direction. In this sense, the appearance of exotic skyrmion state is easy to understand.

Another scenario for the existence of high-order anisotropy terms in B20-type transition-metal silicides and germanides is associated with the weak itinerant-electron ferromagnetism^14^. The outermost electrons in these compounds can be described by an extended Hubbard model on the two-dimensional (2D) lattice. The Hamiltonian consists of the nearest-neighbor natural hopping (*t*_*0*_), SOC-induced hopping (*t*_*SO*_), and Hubbard repulsion (*U*)^15^,^16^,^17^,^18^:


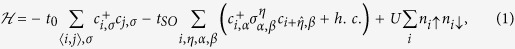


where 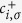 is the operator creating a spin-*σ* (*σ* = ↑, ↓) electron at site *i*, and 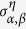 denotes the elements of the Pauli matrix with superscript *η* = *x*, *y*. In the large *U* condition, one can obtain an effective spin Hamiltonian, by a combination of the Heisenberg exchange (*J*), DM interaction (*D*), and quantum compass anisotropy (*A*_*c*_)^15^,^16^,^17^,^18^,^19^:


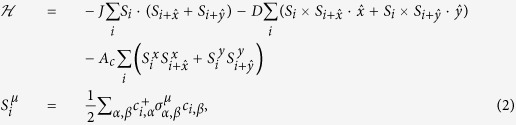


where 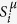 is the spin vector with *μ* ≡ (*x*, *y*, *z*). By defining *t* = (*t*_*0*_^2^+*t*_*SO*_^2^)^1/2^, tan*θ* = *t*_*SO*_/*t*_*0*_, and *J*_*0*_ = 4*t*^2^/*U*, one can obtain *J* = *J*_*0*_ · cos2*θ*, *D* = *J*_*0*_ · sin2*θ*, and *A*_*c*_ = *J*_*0*_ · (1 − cos2*θ*)^16^. For magnetic systems with strong SOC, an easy-plane anisotropy term, i.e. the so-called “compass anisotropy (*A*_*c*_ > 0)”, appears naturally^15^,^16^. This Hamiltonian has recently been used to deal with ultra-cold atoms with artificial SOC on a 2D optical lattice or three-dimensional (3D) counterpart^17^,^18^.

In fact, in the context of solid state physics, hybrid models interpolating between Heisenberg models and compass models are often proposed^20^,^21^,^22^,^23^. Such hybrid models resulting from Hubbard models are relevant to describe the superexchange interactions in transition metal systems with strong SOC, such as those containing 4d and 5d ions Rh, Ru, Os, and Ir, where the effective moments carry both the orbital and spin characters^20^,^22^,^23^. An example can be found in recent experiments demonstrating the coexistence of superconductivity and ferromagnetism on the interfaces between LaAlO_3_ and SrTiO_3_ (2D electron gas)^24^. Interestingly, relevant magneto-transport studies show a strong and gate-tunable Rashba SOC for these 2D conduction electrons, arising from the broken inversion symmetry at the interface^25^. Subsequently, the microscopic mechanism for the interfacial magnetism using a microscopic model (derived from an extended Hubbard model with Rashba SOC) was investigated^15^,^16^. Both the numerical and analytical calculations predict a long-wavelength spiral ground state with a SOC-dependent pitch under zero magnetic field, while hedgehog-like skyrmions appear over a broad range of magnetic field. While the relevant mechanisms for these emergent phenomena have been disputed, the possible role of the compass anisotropy thus becomes interested to some extent^15^,^16^,^17^,^18^,^20^,^22^.

From the viewpoint of energy scale, the compass anisotropy is usually ignored in the literature, since it is an even higher order than the DM term which is already insignificant. We discuss this exceptional issue from several levels:

(1) First, considering a chiral magnet with commonly weak SOC (*t*_*SO*_/*t*_*0*_ ≪ 1), the contribution of compass anisotropy term to the total energy is on an order of magnitude of *A*_*c*_ ~ *J* · (*t*_*SO*_/*t*_*0*_)^2^ which is much smaller than the DM term *D* ~ *J* · (*t*_*SO*_/*t*_*0*_) ≪ *J*^16^. However, MnGe has strong SOC and this compass anisotropy term can no longer be trivial and must be taken into account, for its contribution to the energy is comparable to the DM term (~*D*^2^/*J*)^15^,^16^.

(2) Second, the effect of uniaxial anisotropy *A*_*s*_(*S*_*i*_^*z*^)^2^ on the helical state and SkX in B20-type helimagnets was once studied^26^. In phenomenological sense, this uniaxial anisotropy arises from the single-ion or dipolar shape anisotropy, and thus can be either an easy-axis (*A*_*s*_ < 0) or hard-axis (*A*_*s*_ > 0) anisotropy^27^. To this stage, the effective anisotropy in these materials is governed by *A* = *A*_*c*_ + *A*_*s*_. In some cases, the uniaxial anisotropy is vanished or much smaller than the compass term, which is typically true in MnGe and those magnetic systems with strong SOC mentioned above^15^,^16^. This implies that the compass anisotropy can be a dominant ingredient in the effective anisotropy term in these cases, which has not been well recognized earlier^11^,^13^,^15^,^16^.

(3) Third, it is noted that the anisotropy ingredients would possibly be sensitive to external stimuli. While the uniaxial anisotropy can be tuned by strain^27^, the compass anisotropy may be modulated largely too by external stimuli (e.g. external electric field), as motivated by the tunable SOC in 2D electron gas systems^28^,^29^. These allow possibilities to explore the spin states in chiral magnets with tunable *A* (either uniaxial or compass or both). Obviously, along the line supported by the above three-level discussion, one has sufficient reasons for a theoretical study on the helimagnets with compass anisotropy like MnGe.

In this work, we investigate the spin ground states in such chiral magnets with multifold interactions, using Monte Claro (MC) simulation^30^. Specific attention is paid to the roles of the compass anisotropy. We first explore the phase diagram in the (*H*_*z*_, *A*)-space, which demonstrates the roles of the compass anisotropy in modulating the spin states. Then, we suggest an intriguing alternating half-skyrmion (HSk) and half-anti-skyrmion (HASk) crystal structure (i.e., a state in the phase diagram) to be a candidate for the zero-field ground state of MnGe compound, confirmed by the quite good qualitative consistence of the MC simulations with experiments. We believe that this study provides a theoretical guide to understand the magnetic structures and their evolutions in those helimagnets with strong SOC.

## Results

### Model and Simulation

Considering the helical and skyrmion spin structures in helimagnets MnGe, MnSi, Fe_1−*x*_Co_*x*_Si etc, it does not lose a generality to start from a 2D *L* × *L* square lattice with periodic boundary conditions. One Heisenberg spin *S*_*i*_ is imposed on each site *i*. The Heisenberg exchange interactions, DM interactions, compass anisotropy terms, and Zeeman term are considered. The Hamiltonian Eq. (2) is thus re-written as:


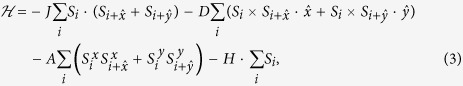


where field *H* is along the *z*-axis or –*x*-axis or stated elsewhere. The uniaxial anisotropy favors the hard-axis along the *z*-axis at *A*_*s*_ > 0, or the easy-axis along the *z*-axis at *A*_*s*_ < 0. Here, *A*_*s*_is a minor quantity in comparison with *A*_*c*_. In this sense, one may reckon that the synthetic anisotropy term (*A*) still prefers the in-planar 90° compass-type symmetry. We will simply treat this synthetic easy-plane anisotropy as the compass anisotropy hereafter.

In the classical approximation, we treat spins *S*_*i*_ as classical vectors and aim at minimizing the energy for finding the ground-state spin configurations {*S*_*i*_}. It is noted that the classical MC simulation has been used to explore the phase diagrams of effective spin models in the similar context, e.g. chiral magnets^8^,^31^, and even ultra-cold atomic systems^17^,^18^. Therefore, we restrict our discussion on the classical approximations, while a full quantum treatment would be needed to have a deeper understanding of quantum spin models, which is beyond the present work.

For simplicity, we use dimensionless units for convenience: spatial length is scaled by the lattice spacing *a*. Parameters *D*, *A*, and *H* are quantified in the units of *J*/*a*, *J*, *J* · *S*, respectively. The spin moment *S* ≡ 1 and *a* = 1 are taken without loss of generality. In this case, the spin phase texture is determined by ratios between *D*, *A*, and *H*. In the simulation, we choose ratio *D*/*J* = √6, yielding the helical wavelength *λ* ~ 8*a* for the spin structure in MnGe. Note that the typical helical wavelength *λ* is ~4.0 nm for MnGe, and the lattice constant *a* is *~*0.48 nm^32^, which is on the same order of magnitude as that of the helical states obtained in our simulations. The simulation algorithm and details of the calculation procedure are presented in the Method section below.

The as-simulated spin structures are characterized by several physical quantities, as suggested in literature^31^. First, the spatial symmetry of spin structure is reflected in the Bragg intensity pattern |*S*(*q*)|^2^, with reciprocal momentum *S*(*q*) obtained from the Fourier transformation of the spin configuration:


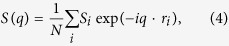


where *N* = *L*^2^ is the total number of spins and *r*_*i*_ is the spatial coodinate.

To characterize swirling structure of the skyrmions lying on the *xy*-plane, we introduce the local density of skyrmions *χ*_*i*_ at lattice site *i*, defined as:


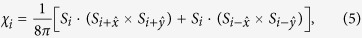


which is the discretization counterpart of the skyrmion density *S*·(∂_*x*_S × ∂_*y*_S)/4π for the continuum case^4^. The summation of *χ*_*i*_ over the extended coordinate space gives the so-called topological winding number (skyrmion number) as *χ* = ∑_*i*_*χ*_*i*_, which is proportional to the topological Hall resistivity 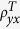 . One single skyrmion cell contributes one unit to *χ* in the continuum limit.

The helicity of a skyrmion state or a helical state is closely related to the spin chirality via the relativistic SOC. We define the helicity as^33^:


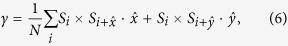


where the sign and magnitude of *γ* reflect respectively the spin swirling direction (i.e., left or right handedness) and degree of swirl in a proper screw spin structure.

Moreover, we define the out-of-plane magnetization, in-plane magnetization, and total magnetization, respectively as:


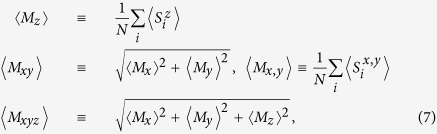


where <…> refers to the thermal and configuration averaging at a given temperature *T*.

### Low temperature phase diagram

**Figure 1:**
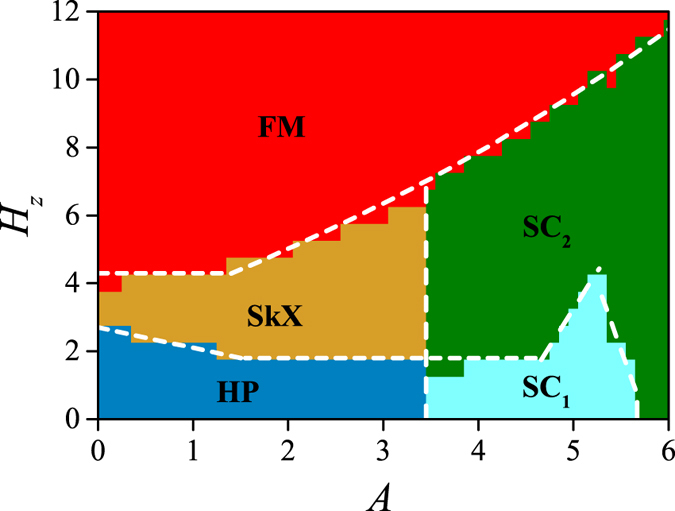
Low temperature (*T* = 0.01) phase diagram of the spin model in Eq. (3) with *D* = √6*J*, and *H* along the *z*-axis (*H*_*z*_). Spin configurations are abbreviated as SkX (skyrmion crystal), HP (helical phase), SC (spin crystal), and FM (ferromagnetic) phase. Two types of spin crystals are identified as SC_1_ and SC_2_ according to their different symmetry. Phase boundaries are marked by white dashed lines.

**Figure 2:**
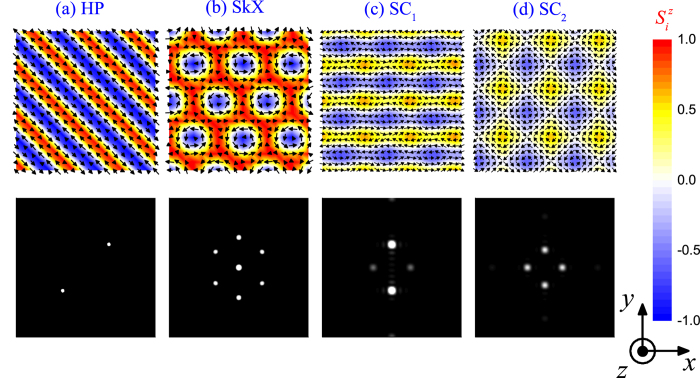
Plot of some typical spin configurations arising in the phase diagram (Fig. 1): (**a**) HP state at (*A*, *H*_*z*_) = (0.2, 0.0), (**b**) SkX phase at (*A*, *H*_*z*_) = (0.2, 2.6), (**c**) SC_1_ phase at (*A*, *H*_*z*_) = (4.2, 0.0) and (**d**) SC_2_ phase at (*A*, *H*_*z*_) = (6.0, 0.0), with the corresponding Bragg intensity |*S*(*q*)|^2^ patterns shown at the bottom.

Extensive simulations over a broad region on the (*A*, *H*_*z*_) plane generate a low temperature phase diagram, as summarized in Fig. 1. Here *H*_*z*_ denotes the field *H* along the *z*-axis. Some typical spin structures of these phases are presented in Fig. 2. To illustrate the spin configurations, we use the color map to scale the spin components along the *z*-axis (out-of-plane) *S*_*i*_^*z*^, and use the spin vectors (arrows) to describe the on-plane *xy* components *S*_*i*_^*xy*^.

For very low *H*_*z*_ ~ 0 in Fig. 1, the result shows the spin structure transition from helical phases (HP) to spin crystal (SC) phases, as the anisotropy *A* increases from zero. Upon applying of an intermediate *H*_*z*_, the HP and SC_1_ phase evolve respectively into the SkX phase and SC_2_ phase, as shown in Fig. 2(b,d). At high *H*_*z*_, the lattice gradually gives away to the FM phase, with all spins orientating along the *z*-axis. This phase transition sequence is consistent with the one in previous studies^31^.

In Fig. 2(c,d), one may find from the Bragg patterns that these crystal structures are constructed primarily from the superimposition of two helices, one oriented along the *x*-axis and the other along the *y*-axis, as characterized by two pairs of Bragg peaks. Here, the equality of these two pairs of Bragg intensities can be used as a criterion to classify the chiral crystal states: the SC_1_ phase (unequal intensities) and SC_2_ phase (equal intensities), following earlier studies^31^. Therefore, the increasing compass anisotropy leads to the transformation from single-*q* spin structures (i.e. HP) to double-*q* spin structures (i.e. SC phases). In the following of this work, we emphasize in the SC phases and investigate their topological properties and magnetic-field-induced evolution behaviors.

### The SC_2_ phase: candidate for the ground state of MnGe

Now we investigate the spin crystal phases, i.e. the SC_1_ and SC_2_ phases shown in Fig. 2(c,d). These spin crystal phases were proposed theoretically^34^, and they have been confirmed by the recent experimental observations on MnGe^11^,^13^. The neutron scattering revealed the multiple-*q* helix structure in MnGe in response to increasing *H*, with the spin helix *q* vectors locked along the <100> direction due to an additional anisotropy originating from the SOC. More importantly, given a square or cubic SkX structure, the Berry phase calculations fit quite well the observed *H*-dependent topological Hall resistivity. In contrast, if a hexagonal SkX structure in MnSi and Fe_1–*x*_Co_*x*_Si is assumed, the calculations fail to fit the observed topological Hall resistivity as a function of *H*. Therefore, the MnGe most likely prefers the square or cubic SkX structures rather than a hexagonal SkX structure^13^.

The square SkX structure or the 2D counterpart of cubic SkX structure is just the SC_2_ phase presented in Fig. 2(d). However, knowledge on microscopic mechanism for the possible SC_2_ phase in MnGe is still lacking^13^. The present work proposes that it is just the compass anisotropy originating from the SOC responsible for formation of the SC phases, noting again that MnGe has strong SOC. To proceed, we start from a SC structure on a 2D plane, which can be viewed as a superimposition of two single helical states^34^:


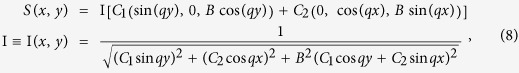


where I ≡ I(*x*, *y*) is a normalized factor, *B* (0 < *B* ≤ 1) is a variable associated with the compass anisotropy term *A*, which stands for the compression of the helical ordering along the *z*-axis brought about by the easy-plane compass anisotropy term. *C*_1_ and *C*_2_ represent the specific weight of the two helices, as characterized by the relative intensities of the two pairs of the Bragg spots shown in Fig. 2(c,d).

### The SC_1_ and SC_2_ phases as the ground states at H~0

**Figure 3:**
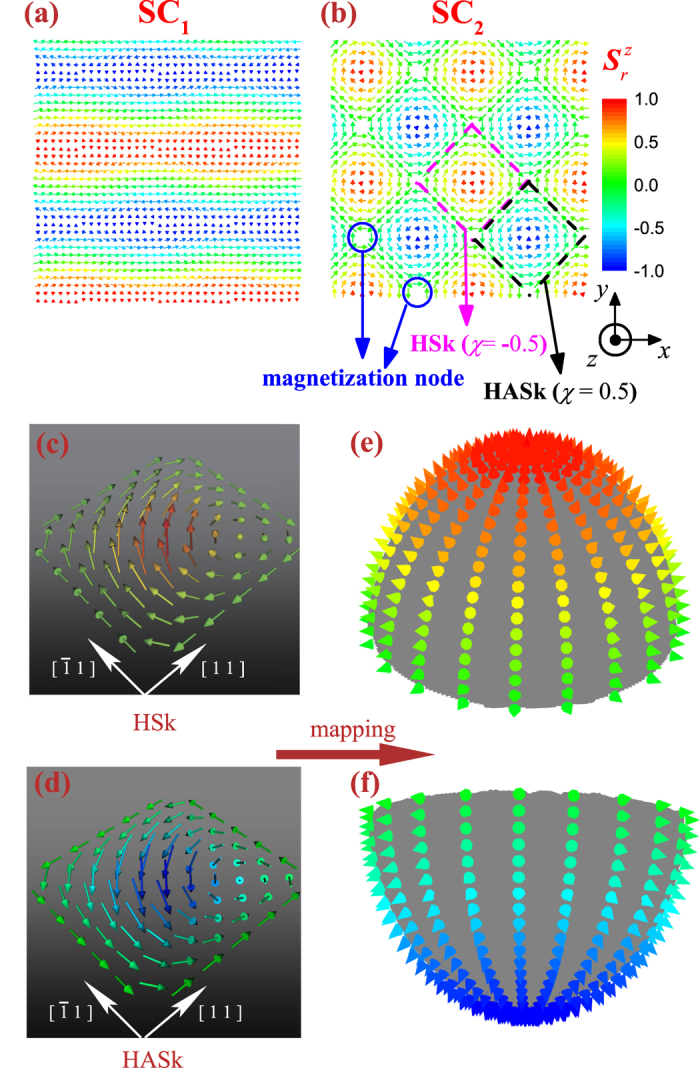
Plots of (**a**) SC_1_ structure given by the expression Eq. (8) with specific parameters *q* = 2π/30, *C*_1_ = 0.8, *C*_2_ = 0.2, and *B* = 0.9; and (**b**) SC_2_ structure with *B* = 0.5, where we find the profile of plotted SC_2_ state with the parameter *B* = 0.5 gives a good fit with that obtained from the MC simulation. A primitive cell in the SC_2_ structure is composed by a HSk structure and a HASk structure, which are enclosed by the dark and pink dash lines, respectively. (**c,d**) Schematic illustrations of the spin configurations in a HSk and a HASk structure in the SC_2_ structure, respectively. (**e,f**) Schematic mapping from a HSk or a HASk spin structure to a hemisphere of the hairy sphere.

We first study the spin structures and possible topological properties of the SC_1_ and SC_2_ phases under *H* = 0, corresponding to the Eq. (8) with *C*_1_ ≠ *C*_2_ and *C*_1_ = *C*_2_, respectively. The typical spin configurations of the two phases are plotted in Fig. 3(a,b) respectively. While the SC_1_ phase is relatively trivial, we concentrate on the SC_2_ phase. We find a periodic array of “nodes” (magnetization nodes) in the SC_2_ phase, where the spin vector *S*_*i*_ = 0^35^. These singular points can be easily seen from Eq. (8) if one sets (sin*qx* = 1, cos*qy* = −1) or (sin*qx* = −1, cos*qy* = 1). The hard-spin constraint *|S*_*i*_*|* = 1 are used in the simulation, where the singularities are naturally forbidden. In fact, however, it is possible that the spins in real chiral magnets are soft due to their interactions with the metallic host or the averaging over fast fluctuations, leading to the possible points of vanishing magnetization. The electronic structure of this nodal topological lattice had been studied recently^36^. However, we find that the magnetic nodes don’t impose substantial impact on the field dependent behaviors and topological properties of the SC_2_ phase, and here we no longer give more discussion on the nodes. The SC_2_ phase consists of alternative alignment of two square flux units, with two specific units enclosed by the dash lines in Fig. 3(b). We calculate the topological winding number *χ* of such two units by adopting a numerical procedure. First, the 2D lattice is discretized on a square mesh with grids up to *N *× *N*, and then the *χ*_*i*_ at each grid point is calculated using Eq. (5). Second, all the *χ*_*i*_ over the defined region are summed, where an enough big *N* is taken so that the error is on the order of 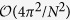 37.

The numerical results show *χ* = 1/2 for one flux unit, and *χ* = −1/2 for another one. Note that the calculated results don’t depend on factor *B* within 0.0 < *B* ≤ 1.0. The topological winding number *χ* = 1/2 or −1/2 for the square flux indicate that this unit is a half-anti-skyrmion (HASk) structure or a half-skyrmion (HSk) structure, with the schematic spin configurations shown in Fig. 3(c,d), respectively. Therefore, the SC_2_ phase at *H* ~ 0 is a spin crystal structure consisting of alternating HSk and HASk units.

It is known that a skyrmion can be topologically mapped into a hairy sphere (or a hedgehog). The vortex-like skyrmion texture found in MnSi and Fe_1–*x*_Co_*x*_Si is topologically equivalent to such a hairy sphere^38^. Applying this topology property to the SC_2_ phase here, one may unfold the HSk and HASk units and map them respectively onto the upper hemisphere and lower hemisphere of the hairy sphere, as shown in Fig. 3(c)~(f). In the other words, the HSk and HASk units can be obtained from splitting a skyrmion into two halves. In this sense, the compass anisotropy causes the fractionalization of the skyrmions.

### Evolution of the SC_2_ phase in response to H_z_

**Figure 4:**
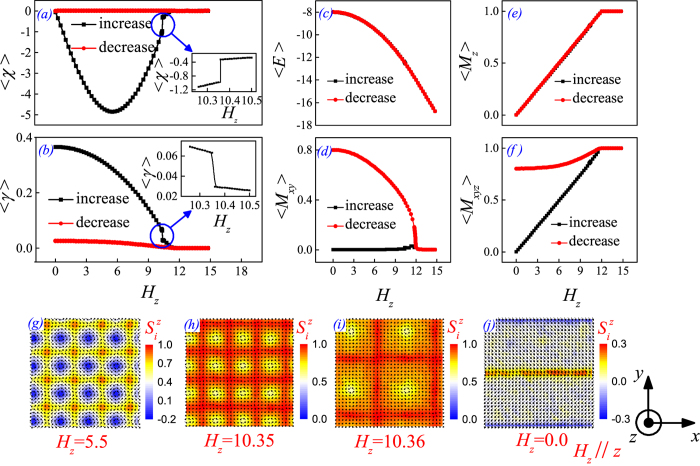
Plots of various physical quantities as a function of *H*_*z*_: (**a**) *<χ>*, (**b**) <*γ*>, (**c**) thermal-averaged energy per spin <*E*>, (**d**) <*M*_*xy*_>, (**e**) <*M*_*z*_>, and (**f**) <*M*_*xyz*_>. Here *<χ>* denotes the thermal average of skyrmion number counted on the spin lattice, and *<γ>* denotes thermal average of the helicity per spin. In this process, spin configurations evolve from the initial SC_2_ state at *H* = 0 to a saturated FM phase driven by the increasing *H*_*z*_, with the state carrying the negative maximal value of *χ* at *H*_*z*_ = 5.5 shown in (**g**). Also, an abrupt expansion of the size of SC_2_ structure is observed at *H*_*z*_~10.3, presented in (**h,i**). Hereafter, a zigzag spin pattern grows with decreasing *H*_*z*_ gradually down back to zero, with the zigzag spin pattern at *H*_*z*_ = 0 shown in (**j**).

Subsequently, we investigate the evolution of the SC_2_ phase in response to the cycling of *H*. To compare with experiments^11^,^13^, we focus on the cases of *H* along the *z*-aixs (*H*_*z*_) and –*x*-axis (*H*_*−x*_). All the simulations start from the initial lattice which is equilibrated at *H* = 0, *D* = √6, *A* = 6.0, and *T* = 0.01, corresponding to the zero-field cooling in experiments. A series of parameters characterizing the SC_2_ phase in response to increasing *H*_*z*_ are plotted in Fig. 4. Here the *H*_*z*_ is varying gradually from zero to a value big enough for saturating the FM phase and then back to zero.

We first look at the evolutions in the *H*_*z*_-increasing half loop. Figure 4(a) shows that the thermal-averaged <*χ*> gradually drops from zero to a negative maximal of −5.0 at *H*_*z*_ = 5.5, implying a broken balance of the *χ*-contributions from the HSk and HASk units in the SC_2_ phase. In the simulation, it is found that the positive skyrmion density in the HSk units reduces markedly, leading to a negative net spin chirality, with the spin structure at *H*_*z*_ = 5.5 shown in Fig. 4(g). Further increasing of *H*_*z*_ drives the <*χ*> back and eventually to die away at *H*_*z*_ ~ 12.0 (corresponding to the FM state). The evolution from the SC_2_ phase to the FM state is not always continuous, but featured with a sharp jump in the <*χ*>-*H*_*z*_ curve at *H*_*z*_ ~ 10.3, accompanied with an abrupt expansion of the HASk unit size and also abrupt jump of the SC periodicity. The spin structures right below and above this jump point are presented in Fig. 4(h,i). Similar phenomenon was also observed in the melting of hexagonal SkX structure. In this process, the increasing *H*_*z*_ destructs the thermal-averaged helicity <*γ*> of the SC_2_ phase, as seen clearly by the <*γ*>-*H*_*z*_ curves shown in Fig. 4(b).

Then we look at the *H*_*z*_-decreasing half loop. Different from the *H*_*z*_-increasing half loop, the lattice returns back to a spin structure aligned along the <10> direction rather than the SC_2_ phase, as shown in Fig. 4(j). This spin structure is characterized by a zigzag pattern with striped domain of alternating *M*_*z*_. The simulations on an extended 64 × 64 lattice confirm the existence of the zigzag striped domains. It is noted that no thermal hysteresis effect appearing in the evolution of magnetic state during the process of increasing and decreasing the magnetic field, as shown by the simulated <*E*>-*H*_*z*_ loop in Fig. 4(c). Therefore, the different magnetic states emerging during this evolution sequence suggest that these states are basically degenerate. Typically, the zigzag striped domains and the SC_2_ phase at *H*_*z*_ = 0 have the same energy. The zigzag spin pattern may align along one of <10> directions on the *xy*-plane at random. Considering the zigzag-patterned spin structure which aligns along the *y*-axis shown in Fig. 4(j), the average *x* component <*M*_*x*_> of magnetization is ∼0, leaving the nonzero <*M*_*y*_>. However, the values of <*M*_*x*_> and <*M*_*y*_> reverse for the zigzag pattern aligned along the *x*-axis. Therefore, we use in-plane magnetization <*M*_*xy*_> rather than <*M*_*x*_> and <*M*_*z*_> to characterize the formation of zigzag-patterned spin structure upon decreasing *H*_*z*_. As shown in Fig. 4(d)~(f), the simulated <*M*_*xy*_>-*H*_*z*_, <*M*_*z*_>-*H*_*z*_, and <*M*_*xyz*_>-*H*_*z*_ curves all confirm that the zigzag spin structure with magnetization on the *xy*-plane develops gradually upon decreasing *H*_*z*_, resulting in the nonzero total magnetization <*M*_*xyz*_> of this spin pattern.

The above simulated results find qualitative consistence with recent experimental observations. First, the simulated <*χ*>-*H*_*z*_ loop is in perfect agreement with measured 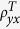 -*H*_*z*_ loop on polycrystalline MnGe at low temperature (5 K), noting that the topological Hall resistivity 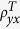 is proportional to *χ* (see Fig. 2(d) in [11]). In addition, the measured <*M*>-*H*_*z*_ loop does show a linear behavior (see Fig. 4(a) in [11]), consistent with the simulated <*M*_*z*_>-*H*_*z*_ dependence. Considering the random distribution of zigzag-patterned spin structure or SC_2_ domains in the *xy*-plane for polycrystalline MnGe, one may compare the simulated <*M*_*z*_>-*H*_*z*_ curves with the measured <*M*_*xyz*_>-*H*_*z*_ curves. Indeed, they coincide with each other quite well.

The *H*_*z*_-driven evolution from the SC_2_ phase to the FM phase can be qualitatively explained, according to a theoretical formula^34^:


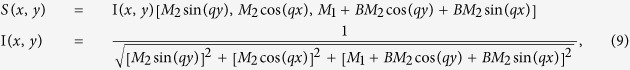


where *M*_1_ denotes the net magnetization induced by *H*_*z*_ and *M*_2_ represents the magnetization of the SC_2_ phase, factor *B* (0 < *B* ≤ 1) again stands for the compression of the helical ordering along the *z*-axis induced by the easy-plane compass anisotropy.

**Figure 5:**
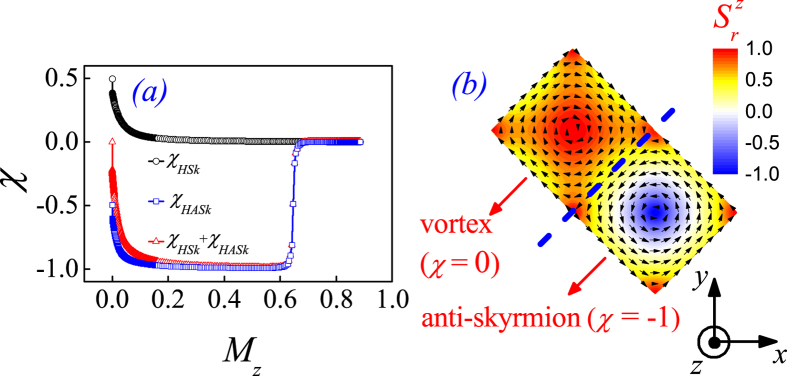
(**a**) A plot of the *χ*-*M*_*z*_ dependence for characterizing the evolution of the SC_2_ phase in response to *H*_*z*_, according to Eq. (9) with *B* = 0.5 chosen here. *χ* is counted in the region of a HSk (HASk) structure, denoted as *χ*_*HSk*_ (*χ*_*HASk*_). (**b**) A typical spin configuration at *M*_*z*_ = 0.55 is shown.

To proceed, we separate one HSk-HASk pair into two independent primitive cells. Then, we discretize each of the HSk unit and HASk unit on a 2D mesh with up to 200 × 200 grids for calculating their topological winding number *χ*_*HSk*_ and *χ*_*HASk*_, as the *χ*-*M*_*z*_ dependences plotted in Fig. 5(a). Although the field *H*_*z*_ is not contained explicitly in Eq. (9), we may use the *χ*-*M*_*z*_ dependence to characterize the *χ*-*H*_*z*_ dependence, by considering that *H*_*z*_ can be scaled by *M*_*z*_ as *M*_*z*_ increases along with *H*_*z*_. We can see that the *χ_HSk_* drops steeply from 0.5 to 0.0 and the *χ*_*HASk*_ from −0.5 to −1.0 with increasing *M*_*z*_ from 0.0 to ~0.2, implying a sharp transformation from the HASk to an anti-skyrmion with *χ* = −1 and from the HSk to a flux (vortex) state with *χ* = 0. Consequently, a nonzero net spin chirality over the whole *xy*-plane emerges, as the typical spin structure at *M*_*z*_ = 0.55 shown in Fig. 5(b). Further increasing *M*_*z*_ from ~0.2 drives the SC_2_ phase to deform continuously and eventually the anti-skyrmion state is melt, resulting in *χ*_*HASk*_ ~ 0.0 at *M*_*z*_ ~ 0.65. The spin chirality becomes zero, corresponding to the FM phase.

To this stage, our calculations suggest the evolution sequence from the SC_2_ phase to the FM phase upon increasing *H*_*z*_ as: (1) HASk unit → anti-skyrmion → flux state/vortex with zero net spin chirality → FM state; (2) HSk unit → flux state/vortex with zero net spin chirality → FM state. Likewise, when *H* is applied along the –*z*-axis, a transition from a HSk unit to a skyrmion is expected.

### Evolution of the SC_2_ phase in response to H_−x_

**Figure 6:**
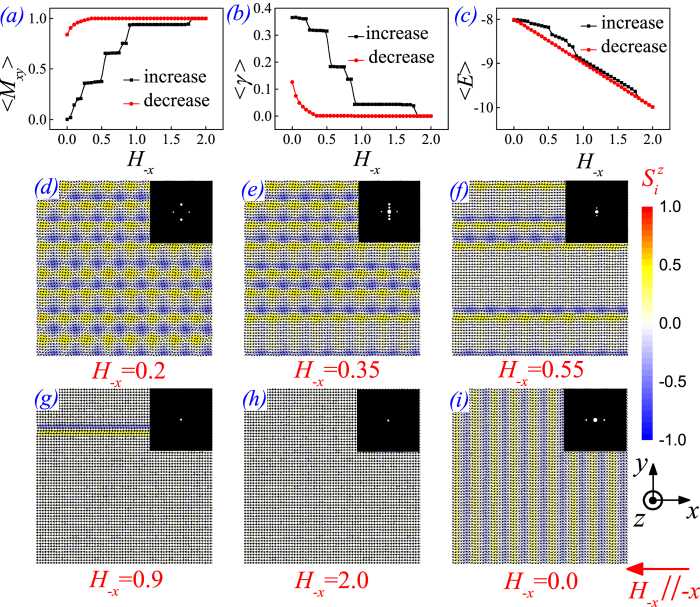
Plots of various physical quantities as a function of *H*_−*x*_: (**a**) <*M*_*xy*_>, (**b**) <*γ*>, and (**c**) energy per spin <*E*>. In this process, spin configuration evolves from the initial SC_2_ state at *H* = 0 to a saturated FM phase driven by the increasing *H*_−*x*_, with some typical states shown in (**d**)~(**h**). Hereafter, a spiral spin pattern grows upon decreasing *H*_−*x*_ gradually down back to zero, with the spiral state at *H*_−*x*_ = 0 shown in (**i**). The upper insets in (**d**)~(**i**) are the corresponding Bragg intensity |*S*(*q*)|^2^ patterns for each spin configuration.

As a complimentary to the last section, we also try to uncover the evolution of the SC_2_ phase in response to an in-plane magnetic field, e.g. along the –*x*-axis (*H*_*−x*_). The main simulated results are summarized in Fig. 6. Here, we perform the MC simulation on a 64 × 64 lattice. Such a big size of lattice makes it better to distinguish the spin structures, and especially the periodicity of these structures. In Fig. 6(a), the simulated <*M*_*xy*_>-*H*_−*x*_ dependence in the increasing *H*_−*x*_ sequence exhibits the multi-step magnetization. The alternating HSk and HASk crystal structure gradually decays into the FM phase with spin aligned along the –*x*-axis, as some typical spin lattices shown in Fig. 6(d)~(h). The periodicity of the spin patterns can be measured from the Bragg intensity patterns in the upper insets, which are associated with the experimental SANS patterns^13^.

In fact, every magnetization step corresponds to a specific spin pattern. At the low-*H*_−*x*_ case, shown in Fig. 6(d), the SC_2_ structure is slightly deformed from the *C*_4_ symmetry, reflected in the two pairs of Bragg spots with different brightness. It should be noted that the experimental ring-shaped SANS pattern, formed in the polycrystalline powder, is generated by the randomly oriented multiple-*q* structures with the same magnitude of magnetic modulation vector (see Fig. 3(a) in [13]). Therefore, the ring pattern is associated with the formation of a periodically modulated magnetic structure (i.e., the SC_2_ phase here). Further increasing *H*_−*x*_ triggers some stripe-like domains along the *x*-axis shown in Fig. 6(e) at *H*_−*x*_ = 0.35. These stripes are mixture of the alternating HSk and HASk crystal structure and the FM state aligned along the –*x*-axis with inharmonic periodicity. The corresponding Bragg intensity pattern shows a pair of Bragg spots in the *q*_*x*_**-axis and two pairs of Bragg spots in the *q*_*y*_**-axis, in good agreement with the crescent-shaped SANS pattern observed experimentally (Fig. 3(b) in [13]). At very high *H*_−*x*_, the Bragg spots shrink and the lattice gradually gives away to the FM phase, also consistent with experiments^13^. Similar features can be found in the <*γ*>-*H*_−*x*_ curve (Fig. 6(b)). When *H*_−*x*_ is gradually ramped down back to zero, a spiral state emerges at *H*_−*x*_ ~ 0.3. As shown in Fig. 6(i), the spiral propagates along the –*x*-axis with a good periodicity, as the variation in the Bragg patterns again shows good consistence with the variation of SANS patterns (see Fig. 3(e)~(h) in [13]).

It is noted from the <*E*>-*H*_−*x*_ curves in Fig. 6(c) that, the spiral state is degenerate with the alternating HSk and HASk crystal structure at *H*_−*x*_ = 0. However, energy of the states formed in the process of increasing *H*_−*x*_ is apparently higher than that in the process of decreasing *H*_−*x*_, at the range of 0 < *H*_−*x*_ < ~1.8. In this regard, we reckon that the alternating HSk and HASk crystal structure is a strongly frustrated spin system^39^. This is why the multi-step magnetization appearing in the <*M*_*xy*_>-*H*_−*x*_ curve in Fig. 6(a).

## Discussions

Before concluding this work, we briefly discuss experimental realization of the SC_2_ phase. B20-type helimagnets with another type of easy-plane anisotropy *A*_*s*_(*S*_*i*_^*z*^)^2^ (*A*_*s*_ > 0) were also studied by MC simulation, which shows that this anisotropy may lead to the alternating HSk and HASk crystal structures on a square lattice at proper *A* too^40^. In addition, skyrmions in B20-type helimagnet thin films with easy-plane anisotropy *A*_*s*_(*S*_*i*_^*z*^)^2^ (*A*_*s*_ > 0) were recently studied using a micromagnetic model including demagnetization and a three-dimensional geometry^41^. This work showed the demagnetization effects and/or the finite thickness effects on the skyrmion energetics and stability, which were explicitly demonstrated in earlier studies^42^,^43^. Therefore, B20-type helimagnet thin films seem to be appropriate for realizing the SC_2_ phase, because the uniaxial anisotropy (*A*_*s*_), compass anisotropy (*A*_*c*_) as well as the demagnetization can be altered significantly by varying the film thickness and the laterally confined geometries^27^,^41^,^42^,^44^. Furthermore, hedgehog skyrmions in chiral magnets with Rashba SOC were previously studied by variational energy calculations, which imposes a topological constraint for the center and boundary spins in a unit cell^16^. This constraint allows the hedgehog skyrmions but naturally exclude the HSk or HASk states in the chiral magnets. Therefore, whether the compass anisotropy can lead to the energetically stable HSk or HASk states in chiral magnets with Rashba SOC is yet to be explored.

On the other hand, temperature dependence of the Hall resistivity, susceptibility and magnetic modulation vector in MnGe was revealed by experiments^11^,^13^. These features are also important for our understanding of the underlying physics of MnGe, which would be an interesting challenge for our further studies.

In summary, we have presented in this work a detailed analysis of a classical microscopic spin model comprising FM exchange, DM interaction, compass anisotropy, and Zeeman energy, which may be widely used for the chiral magnets with strong SOC. We use classical MC simulation to determine the low temperature phase diagram as a function of compass anisotropy and out-of-plane magnetic field. In this phase diagram, we propose the alternating HSk and HASk crystal structures on a square lattice to be the candidate for the zero-field ground state of the helimagnet MnGe. The simulated evolution of the spin structure driven by magnetic field is in good accordance with experimental observations on MnGe. Therefore, our microscopic spin model successfully captures the main physics responsible for the magnetic structures in the helimagnet MnGe. This study may also provide a new theoretical guide to understand the magnetic states in other systems (such as the complex oxide heterostructure interfaces, and artificial ultracold Bose gas) with strong SOC, and may bring some insights for the future experiments.

## Methods

### Simulation algorithm

In this work, we perform MC simulations based on the standard single-flip Metropolis algorithm combined with the over-relaxation method. This clustering algorithm is believed to be effective in equilibrating the frustrated spin models with a large size^45^,^46^,^47^. Our unit MC step consists of one Metropolis sweep and ten over-relaxation sweeps. Most calculations are carried out on a 32 × 32 lattice with periodic boundary conditions, and then further checked on a lattice with bigger size of 64 × 64, in which case the simulation results remain the same. For reaching the ground state, an annealing simulation scheme is employed. First, a paramagnetic lattice is chosen as the initial lattice and each simulation cycle starts from sufficiently high temperature (*T*). Then the lattice is cooled down gradually until a very low *T* = 0.01^8^,^31^. The as-obtained spin state is treated approximately as the ground state.

For tracking the evolution of non-zero field spin states, a ladder protocol following earlier reports^39^,^48^,^49^ is employed. The corresponding zero-field ground state is chosen as the initial lattice, and then magnetic field *H* is varied following the linear protocol *H*(*j*) = *H*(*j* −1) + *δt*, where *H*(*j*) (*j* = 1, 2, 3, …) is the field at stage *j*, *δ* is a constant, and *t* is time measured in MC steps. In the protocol, the lattice at *H*(*j* −1) is taken as the initial lattice for calculating the state at *H*(*j*). Besides, we discard the data in the first *N*_*e*_ MC steps needed for equilibration, and calculate the averages over the following *N*_*c*_ MC steps at every *H*(*j*) stage. Typically, both *N*_*e*_ and *N*_*c*_ are 5 × 10^5^. During this procedure, field *H* is increased from *H* = 0 till a sufficiently big magnitude so that the lattice reaches the field-driven FM state. Subsequently, the field is reduced back to *H* = 0 for exploring potential hysteresis behavior if any.

## Additional Information

**How to cite this article**: Chen, J. P. *et al*. Exotic skyrmion crystals in chiral magnets with compass anisotropy. *Sci. Rep*. **6**, 29126; doi: 10.1038/srep29126 (2016).
